# Warmer environmental temperature accelerates aging in mosquitoes, decreasing longevity and worsening infection outcomes

**DOI:** 10.1186/s12979-024-00465-w

**Published:** 2024-09-11

**Authors:** Jordyn S. Barr, Lindsay E. Martin, Ann T. Tate, Julián F. Hillyer

**Affiliations:** Department of Biological Sciences, Vanderbilt University, Nashville, TN USA

**Keywords:** Climate Change, Survival, Infection Intensity, Insect, Senescence, Mosquito, Physiology

## Abstract

**Background:**

Most insects are poikilotherms and ectotherms, so their body temperature is predicated by environmental temperature. With climate change, insect body temperature is rising, which affects how insects develop, survive, and respond to infection. Aging also affects insect physiology by deteriorating body condition and weakening immune proficiency via senescence. Aging is usually considered in terms of time, or chronological age, but it can also be conceptualized in terms of body function, or physiological age. We hypothesized that warmer temperature decouples chronological and physiological age in insects by accelerating senescence. To investigate this, we reared the African malaria mosquito, *Anopheles gambiae*, at 27 °C, 30 °C and 32 °C, and measured survival starting at 1-, 5-, 10- and 15-days of adulthood after no manipulation, injury, or a hemocoelic infection with *Escherichia coli* or *Micrococcus luteus*. Then, we measured the intensity of an *E. coli* infection to determine how the interaction between environmental temperature and aging shapes a mosquito’s response to infection.

**Results:**

We demonstrate that longevity declines when a mosquito is infected with bacteria, mosquitoes have shorter lifespans when the temperature is warmer, older mosquitoes are more likely to die, and warmer temperature marginally accelerates the aging-dependent decline in survival. Furthermore, we discovered that *E. coli* infection intensity increases when the temperature is warmer and with aging, and that warmer temperature accelerates the aging-dependent increase in infection intensity. Finally, we uncovered that warmer temperature affects both bacterial and mosquito physiology.

**Conclusions:**

Warmer environmental temperature accelerates aging in mosquitoes, negatively affecting both longevity and infection outcomes. These findings have implications for how insects will serve as pollinators, agricultural pests, and disease vectors in our warming world.

**Supplementary Information:**

The online version contains supplementary material available at 10.1186/s12979-024-00465-w.

## Background

Some insects are beneficial to humans by serving as pollinators and food sources, but others are detrimental to humans by destroying agricultural crops and transmitting disease [1]. Mosquitoes are detrimental because they transmit pathogens that kill hundreds of thousands of people every year [2]. To transmit disease, mosquitoes must acquire a pathogen via a bloodmeal, serve as a competent host for that pathogen, outlive the pathogen’s extrinsic incubation period, and pass the pathogen to a vertebrate host via a second bloodmeal [2]. Many factors—including temperature and aging—affect how well mosquitoes survive, overcome, or transmit an infection [3–7].

Global temperatures are rising, exposing insects to warmer temperatures than they are accustomed to [8]. Most insects are poikilotherms and ectotherms, so their body temperature is predicated by the temperature of the environment, which has profound effects on physiology and life history. For mosquitoes, warmer temperature decreases egg hatching rates [9], reduces larval and pupal survival [10, 11], and shortens the adult lifespan [12], all of which lower the number of mosquitoes in a population. However, warmer temperature also accelerates development [13–15], which reduces generation time and may increase the number of mosquitoes available to transmit disease. Finally, warmer temperature increases the metabolic rate [16, 17], reduces adult size [18, 19], and modifies the strength of immune responses in complex ways [20–23], leading to a decline in vector competence [3, 24]. Therefore, changes in temperature can greatly impact the physiology of mosquitoes, altering their ability to be pests and transmit disease.

Aging also shapes insect physiology. Mosquitoes and other insects senesce as they age, which deteriorates their body condition, lowers egg production and the metabolic rate, and weakens immune prowess [7, 25–27]. As immunosenescence progresses, mosquitoes become more susceptible to infection [3]. However, the decline in physiological resources and longevity that occurs with advanced aging can reduce vector competence [6]. Therefore, aging has complex effects on disease transmission dynamics. A recent study in *An. stephensi* showed that when mosquitoes are reared at a standard temperature and then shifted to warmer or cooler temperatures as adults, the sensitivity of some traits changes: the aging-dependent decline in the biting rate and egg production is most pronounced at the warmest temperatures, and uninfected mosquitoes are more likely to die when the temperature is warmer [28]. Therefore, although advanced aging is thought to lower the probability that a mosquito transmits a pathogen [29], changes in environmental temperature may alter the effects of aging.

Aging is traditionally conceptualized in terms of time, or chronological age. However, aging can also be conceptualized in terms of body function and efficiency, or physiological age. Homeotherms, like mammals, use their metabolism to maintain a constant body temperature, so their chronological age and physiological age are intricately linked [30]. Many other animals, including mosquitoes, do not use metabolism to regulate their body temperature, and therefore, their body temperature fluctuates with the temperature of their environment [8, 30]. In mosquitoes, we hypothesize that chronological age and physiological age are unlinked by temperature, with physiological aging occurring faster when the temperature is warmer, and therefore, warmer temperature accelerates the progression of senescence. Recent data support this hypothesis. The interaction between temperature and aging impacts the body composition of the adult mosquito: warmer temperature accelerates the decline in protein content that occurs with aging [18]. Additionally, warmer temperature accelerates the aging-dependent weakening of the melanization immune response [31]. Therefore, we predict that temperature and aging interact to shape vital facets of mosquito physiology.

Using the African malaria mosquito, *Anopheles gambiae*, we examined how mosquito survival is impacted by a hemocoelic bacterial infection, warmer environmental temperature, aging, and the interaction between temperature and aging. Our findings demonstrate that (i) mosquito longevity declines in the presence of a bacterial infection in the hemocoel, (ii) mosquitoes have shorter lifespans when the temperature is warmer, (iii) older mosquitoes are more likely to die, and (iii) warmer temperature marginally accelerates the aging-dependent decline in survival. Furthermore, we investigated how the intensity of an *E. coli* infection changes with warmer temperature and mosquito aging. We discovered that infection intensity increases when the temperature is warmer and with aging, and that warmer temperature accelerates the increase in infection intensity that occurs with aging. By repeating the experiment in mosquitoes reared at three temperatures but infecting them all at a single temperature, we uncovered that the changes in infection intensity that are due to temperature occur because of effects on both mosquito and bacterial physiology. In summary, temperature uncouples chronological and physiological aging, and specifically, warmer temperature accelerates senescence. Because most insects are poikilothermic ectotherms, these findings have implications for how insects serve as pollinators, agricultural pests, and disease vectors.

## Methods

### Mosquito rearing and colony maintenance

*Anopheles gambiae*, Giles sensu stricto (G3 strain; Diptera:Culicidae) were reared as previously described [18, 31]. Briefly, a colony of mosquitoes was maintained at 27℃ and 75% relative humidity with a 12 h:12 h light:dark cycle. The colony was fed 10% sucrose solution ad libitum and fed defibrinated sheep’s blood (Hemostat Laboratories Inc., Dixon, CA) weekly using an artificial membrane feeder (Hemotek Ltd., Blackburn, UK).

**Fig. 1:**
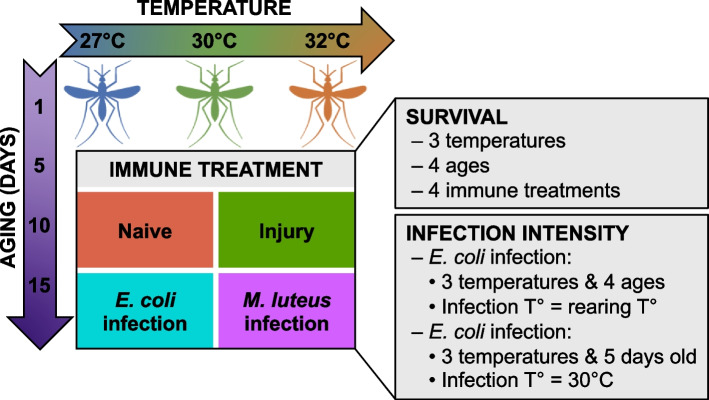
Experimental overview for investigating the effects of warmer temperature, aging, and their interaction on mosquito survival and infection intensity

Eggs were collected 2 days after blood feeding and were split into three environmental chambers, each set to 75% relative humidity, a 12 h:12 h light:dark cycle, and one of three constant temperatures: 27℃, 30℃, or 32℃. These temperatures were selected because they lie within the thermal sensitivity range of the insect and correspond to warming temperatures in nature. Moreover, temperatures warmer than 32℃ significantly reduce egg hatching and survival of the immature stages [4], limiting experimental feasibility. Larvae were fed a mixture of koi fish food and baker’s yeast daily, and when present, pupae were collected daily and transferred to plastic containers with fine mesh netting. After eclosion, adults were maintained on a 10% sucrose diet. Experiments were conducted on female adults at 1, 5, 10, and 15 days post-eclosion that had been reared and maintained at each temperature. These ages were selected because they span the lifespan of the adult mosquito—immediately after eclosion, to reproductively mature, to advanced age. Female mosquitoes were held with male mosquitoes until experimentation, at which point the males were discarded. This experimental design captures the effects of temperature throughout the entire life of the mosquito and not just the effects of temperature on the adult stage (Fig. 1).

### Mosquito survival: four immune treatments at three temperatures and four ages

For each temperature-age combination, mosquitoes were divided into four immune treatments: naïve (unmanipulated), injured (injected sterile LB), infected with *Escherichia coli* (Gram-negative bacteria; modified DH5α, GFP-expressing and tetracycline-resistant), or infected with *Micrococcus luteus* (Gram-positive bacteria; ATCC 4698). These two bacterial species were selected because they elicit different immune responses in the mosquito [32], and because they have different ideal growing temperatures: 37℃ for *E. coli* and 30℃ for *M. luteus*. Mosquitoes were cold anesthetized for immobilization, and those in the naïve treatment group were transferred into a new container without any further manipulation. Mosquitoes in the injured treatment group were injected at the thoracic anepisternal cleft with 69 nL of sterile LB media using a Nanoject III Programmable Nanoliter Injector (Drummond Scientific, Broomall, PA). Mosquitoes in the infected treatment groups were injected at the anepisternal cleft with 69 nL of either *E. coli* or *M. luteus* culture that was at OD_600_ = 2, which is an approximate dose of 18,000 and 11,000 bacteria, respectively. These doses were selected because they elicit an immune response [31, 33], and a pilot study demonstrated that they permit the study of mosquitoes of broad ages reared at different temperatures. Mosquitoes were then returned to their respective temperatures and their survival was monitored every 24 h until all adults had perished. Throughout the experiment, mosquitoes were given new cotton soaked in 10% sucrose solution daily. Three to five biological trials, each composed of 30–40 mosquitoes, were completed for each temperature-age-immune treatment combination. For this experiment, survival was measured for 8,445 mosquitoes.

Kaplan–Meier survival curves were graphed using GraphPad Prism version 9.5.1, and the data were analyzed using R statistical software version 4.3.2 [34]. Survivorship is described using 3 terms: hazard ratio, median survival, and maximum lifespan. The hazard ratio, shown in forest plots, measures how immune treatment, temperature, aging, or the interaction between temperature and aging contributes to a greater or lesser hazard to survival when compared to the naïve immune treatment, coolest temperature, or the youngest age. Median survival is the day when half the mosquitoes had perished, and maximum lifespan is the day when the longest living mosquito perished.

To determine how immune treatment impacts longevity, the survival curves of injured and infected mosquitoes were compared to the survival curve of naïve mosquitoes. To determine how warmer temperature impacts longevity, the survival curves of mosquitoes reared at 30℃ and 32℃ were compared to the survival curve of mosquitoes reared at the coolest temperature of 27℃. To determine how aging impacts longevity, the survival curves of mosquitoes whose survival monitoring was initiated at 5, 10, and 15 days of age were compared to the survival curve of mosquitoes whose survival monitoring was initiated at 1-day post eclosion. For aging comparisons, the survival data were left-truncated to control for waiting time across age groups because the experiments for the older groups were initiated later in life. Left-truncation aligns the survival curves for the period of time when survival was monitored and removes the assumption that all mosquitoes in a group survived until the monitoring of survival was initiated. All mosquitoes used in the experiments were included in the analyses (no data were discarded), and survival was monitored for the entire lifespan of each mosquito.

Initially, the data were assessed by Cox proportional hazards using the “survival” package in R [35], for the main effects of temperature, age, and their interaction, separated by immune treatment and using experimental block as a random effect. The resulting residuals and log–log plots revealed nonproportional hazards associated with aging in both the *E. coli* and *M. luteus* infection groups, meaning that the data did not meet the assumptions of the Cox proportional hazards model. Therefore, the data were then assessed by Cox regression models with weighted estimation. This method assumes nonproportional hazards and uses the AHR method (“coxphw” package; [36]) to assess how temperature, age, and their interaction impacts survival within each immune treatment.

### Infection intensity: *E. coli* infection at three temperatures and four ages; infection at three temperatures

The *E. coli* infection intensity in septic mosquitoes was determined using a previously described plating method [37]. Briefly, mosquitoes from each temperature-age combination were injected at the thoracic anepisternal cleft with 69 nL of GFP-expressing, tetracycline-resistant *E. coli* at OD_600_ = 2. Mosquitoes were then returned to their respective rearing temperatures and given new cotton soaked in 10% sucrose solution. At 24 h post infection, each mosquito was individually homogenized in 200 µL phosphate buffer saline (PBS), the homogenate was diluted between 1:500 and 1:8000 (depended on the temperature-age combination to allow for the counting of colonies on plates), and the dilution was spread on LB plates containing tetracycline for the selection of live bacteria of the strain introduced into the mosquito during infection. Plates were incubated at 37℃ for 16 h, colony forming units (CFUs) were counted, and the *E. coli* infection intensity for each mosquito was calculated after accounting for the dilution factor. Four to five biological trials, each composed of 10–12 mosquitoes, were completed for each temperature-age combination. For this experiment, infection intensity was measured in 696 mosquitoes. These experiments were only conducted using *E. coli* because our *M. luteus* strain does not contain a selectable marker.

Infection intensity data were graphed using GraphPad Prism version 9.5.1 and analyzed using R statistical software version 4.2.2 [34]. For the analyses, a zero-inflated, negative binomial model (“pscl” package) was used to assess how temperature, age, and their interaction influence infection intensity [38–41]. The final model has two components: (i) a zero-inflated component that uses a binomial distribution with a logit link to predict the probability of a mosquito having cleared an infection (zero CFUs), and (ii) a count component that uses a negative binomial distribution with a log link to predict the number of bacteria in an active infection [38–41]. The final model was determined by stepwise, multidirectional selection from the full model to minimize both log-likelihood values and Akaike Information Criterion (AIC). Goodness of fit was evaluated by comparing the observed and predicted number of zero and non-zero CFU counts. Then, ordinary, non-parametric bootstrapping (“boot” package) was used to identify parameter estimates, 95% confidence intervals, odds ratios (for the zero component), and incidence rate ratios (risk ratios; for the count component) on the multiplicative scale [42, 43]. Finally, the effects of temperature, age, and their interaction were evaluated by comparing the estimated marginal means using the “emmeans” package [44].

### Infection intensity: *E. coli* infection at three temperatures and one age; infection at one temperature

To parse the effects of warmer temperature on mosquito and bacterial physiology, a second infection intensity experiment was conducted where mosquitoes that had been reared at the three temperatures were all infected with *E. coli* at 30 °C. The standardized infection temperature was selected to minimize the temperature shift experienced by mosquitoes reared at 27 °C and 32 °C. Briefly, five-day-old mosquitoes reared at 27℃, 30℃, or 32℃ were injected with GFP-expressing, tetracycline-resistant *E. coli* at OD_600_ = 2. Then, all mosquitoes were placed at 30 °C and given a new cotton soaked with 10% sucrose solution. At 24 h post infection, *E. coli* infection intensity in individual mosquitoes was measured using the plating assay described above. Four biological trials, each composed of 10–12 mosquitoes, were completed for each rearing temperature. For this experiment, the infection intensity was measured in 141 mosquitoes.

Infection intensity data were graphed and analyzed using GraphPad Prism (version 9.5.1). This graphing and analysis included the data from this experiment (see paragraph above) and the data from 5-day-old mosquitoes at each temperature in the initial infection intensity experiment (see prior methods section). Data were assessed for normality and determined to be non-normal. Therefore, data were analyzed using the Kruskal–Wallis, nonparametric test, followed by Dunn’s Multiple Comparison’s post hoc test. All data collected this study is presented in a supplementary file (Additional File 1: File S1).

## Results

### Mosquito survival declines with injury, bacterial infection, warmer temperature, and aging

We first set out to determine how each variable—immune treatment, temperature, aging, and the interaction between temperature and aging—shapes mosquito survivorship, and did so by examining median survival, maximum life expectancy, and the added hazard each variable poses to mosquito longevity (Fig. 1).

**Fig. 2:**
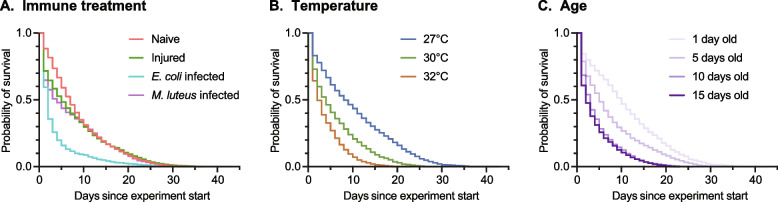
Survival declines with bacterial infection, warmer temperature, and aging. **A** Mosquito survival following different immune treatments, irrespective of temperature or age. **B** Mosquito survival at different temperatures, irrespective of age or immune treatment. **C** Mosquito survival when the experiment is initiated at different ages, irrespective of temperature or immune treatment. The panels plot the same data that is aggregated by different factors. These data are further parsed in Additional File 3: Fig S1, S2 and S3

**Fig. 3:**
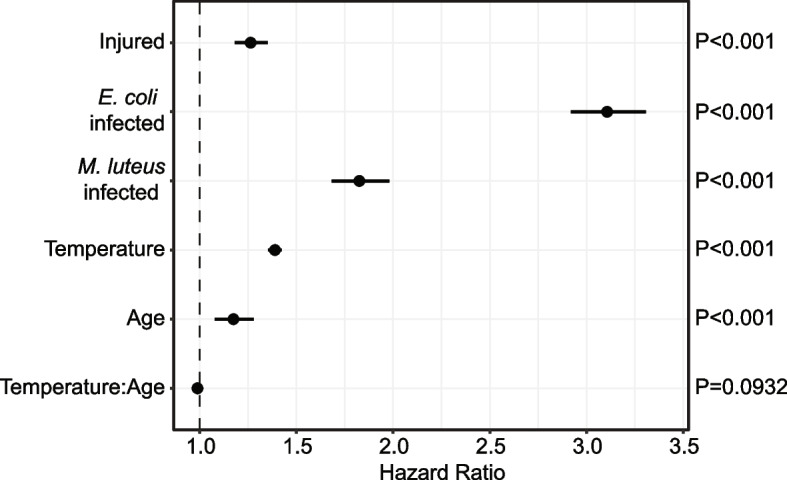
The risk of dying increases with bacterial infection, warmer temperature, and aging. Forest plot shows how immune treatment, temperature, aging, and the interaction between temperature and aging influence the risk of dying. Circles indicate the hazard ratio and lines mark the 95% confidence interval. Hazards greater than 1.0 indicate a greater risk of death. Data were analyzed by Cox nonproportional hazards

To determine how an injury or bacterial infection affects mosquito survival, we measured the longevity of naïve (unmanipulated), injured, and bacterially infected mosquitoes, regardless of the temperature at which they were maintained or the age when the experiment was initiated. As expected, naïve mosquitoes lived the longest (Fig. 2A). When compared to naïve mosquitoes, infection with *E. coli* and *M. luteus* increased the risk of dying by 211% (CI: 192%-231%) and 83% (CI: 68%-98%), respectively, whereas injury increased this risk by 26% (CI: 18%-35%) (Fig. 3 and Additional File 2: Table S1).

To determine how temperature, aging, and the interaction between temperature and aging shape survival, we measured the longevity of mosquitoes reared at 27℃, 30℃, or 32℃ starting at 1, 5, 10, or 15 days post-eclosion, without considering their infection status. Both warmer temperature and aging reduce survival (Fig. 2B and 2C). Specifically, each 1℃ increase in temperature increases the risk of a mosquito dying by 39% (CI: 35%-42%), and each additional day of aging increases the risk of a mosquito dying by 17% (CI: 8–28%) (Fig. 3 and Additional File 2: Table S1). Finally, the interaction between temperature and aging, regardless of immune treatment, did not increase the risk of a mosquito dying (Fig. 3 and Additional File 2: Table S1).

In summary, bacterial infection, temperature, and aging all shape mosquito longevity. Warmer temperature and aging individually reduce mosquito survivorship, but an infection poses the greatest hazard. To gain a deeper understanding of how temperature, aging, and the interaction between temperature and aging shape longevity, we subsequently analyzed the different immune treatment conditions separately.

### The survival of naïve mosquitoes declines with warmer temperature and aging

**Fig. 4:**
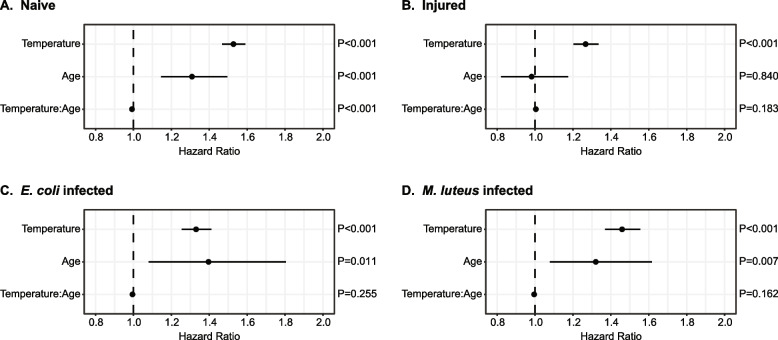
The risk of dying increases with warmer temperature, aging, and their interaction. Forest plots show how temperature, aging, and their interaction influence the risk of dying in naïve mosquitoes (**A**), injured mosquitoes (**B**), *E. coli*-infected mosquitoes (**C**), and *M. luteus*-infected mosquitoes (**D**). Circles indicate the hazard ratio and lines mark the 95% confidence interval. Hazards greater than 1.0 indicate a greater risk of death. Data were analyzed by Cox nonproportional hazards

To determine the baseline effects of rising temperature, aging, and their interaction on mosquito longevity, we analyzed the survival of naïve mosquitoes. Naïve mosquitoes had shorter lifespans when the temperature was warmer, regardless of the age when the experiment was initiated (Fig. 4A and Additional File 3: Fig S1). Specifically, the median survival decreased from 21 days when the mosquitoes were reared at 27 °C to 8 days when reared at 32 °C. Similarly, the maximum lifespan decreased from 38 days at 27 °C to 24 days at 32 °C, and each 1 °C increase in temperature increased the likelihood of a mosquito dying by 53% (CI: 47%-59%) (Fig. 4A, Additional File 3: Fig S1 and Additional File 2: Table S2 and S3).

Naïve mosquitoes that were older were more likely to die compared to those that were younger, regardless of the temperature in which they were reared and maintained (Fig. 4A and Additional File 3: Fig S1). Specifically, one additional day of aging increased the likelihood of a mosquito dying by 31% (CI:15%-50%) (Fig. 4A and Additional File 2: Table S3).

Finally, warmer temperature had a small effect on the aging-dependent decline in survival, and vice versa. For example, the shape of the survival curve of 15-day-old naïve mosquitoes at 27℃ resembles the survival curves of 5-day-old and 1-day-old naïve mosquitoes at 30℃ and 32℃, respectively, indicating that younger mosquitoes reared at the warmer temperatures have similar survivorship to older mosquitoes reared at the cooler temperatures (Additional File 3: Fig S1). Although the interaction between temperature and age was statistically significant, the hazard ratio shows that it has < 1% (CI: -1%-0%) impact on survival, so the effect is small (Fig. 4A and Additional File 2: Table S3). This means that for every 1℃ increase in temperature, an additional day of aging has less than the expected effect of aging alone on the risk of a mosquito dying. This may be because mortality rates saturate as both temperature and age increase toward their maximum. For example, the small interactive effect is eclipsed by the large effect of warm temperature, where mosquitoes died very early in life. In fact, this large effect prevented us from collecting survival data on 15-day-old mosquitoes reared at 32℃. In summary, warmer temperature and aging, and to a small extent their interaction, reduce the survival of naïve mosquitoes.

### The survival of injured mosquitoes declines with warmer temperature

We next assessed how the survival of injured mosquitoes is shaped by warmer temperature, aging, and their interaction. Like for naïve mosquitoes, injured mosquitoes have shorter lifespans when the temperature is warmer, regardless of the age when the experiment was initiated (Fig. 4B and Additional File 3: Fig S2). Specifically, the median survival decreased from 20 days at 27 °C to 7 days at 32 °C, the maximum lifespan decreased from 38 days at 27 °C to 20 days at 32 °C, and each 1 °C increase in temperature increased the risk of an injured mosquito dying by 27% (CI: 20%-33%) (Fig. 4B, Additional File 3: Fig S2 and Additional File 2: Table S2 and S3).

Unlike what we observed for naïve mosquitoes, younger and older mosquitoes, regardless of temperature, had a similar risk of dying following injury (Fig. 4B, Additional File 3: Fig S2 and Additional File 2: Table S3). Furthermore, temperature and aging do not interact to shape the survival of injured mosquitoes (Fig. 4B and Additional File 2: Table S3). Taken altogether, temperature, but neither aging nor the interaction between temperature and aging, shapes the survival of injured mosquitoes.

### The survival of bacterially infected mosquitoes declines with warmer temperature and aging

We then analyzed how the survival of mosquitoes infected with either *E. coli* or *M. luteus* is impacted by warmer temperature, aging, and their interaction. Like for naïve and injured mosquitoes, the survivorship of infected mosquitoes declines when the temperature is warmer, regardless of the age when the experiment was initiated (Fig. 4C and D and Additional File 3: Fig S3). In *E. coli*-infected mosquitoes, the median survival decreased from 9 days at 27 °C to 3 days at 32 °C, the maximum lifespan decreased from 44 days at 27 °C to 16 days at 32 °C, and the risk of dying increased by 33% (CI: 25%-41%) for each 1 °C increase in temperature (Fig. 4C, Additional File 3: Fig S3 and Additional File 2: Table S2 and S3). In *M. luteus*-infected mosquitoes, the median survival decreased from 20 days at 27 °C to 4 days at 32 °C, the maximum lifespan decreased from 37 days at 27 °C to 15 days at 32 °C, and the risk of dying increased by 46% (CI: 37%-55%) for each 1 °C increase in temperature (Fig. 4D, Additional File 3: Fig S3 and Additional File 2: Table S2 and S3). Overall, warmer temperature reduces the longevity of bacterially infected mosquitoes.

When a bacterial infection was initiated at an older age, survival declined faster than when the infection was initiated at a younger age (Fig. 4C and D and Additional File 3: Fig S3). For example, when an *E. coli* infection was initiated at 1-day post-eclosion, fewer than 20% of mosquitoes died within the first 24 h, but when the infection was initiated at 10 days post-eclosion, more than 50% of mosquitoes died within the first 24 h. Each day of aging prior to the initiation of infection increased the risk of dying from an *E. coli* or *M. luteus* infection by 40% (CI: 8%-80%) and 32% (CI: 8%-62%), respectively (Fig. 4C and D and Additional File 2: Table S3). This indicates that a mosquito infected with bacteria at an older age is more likely to die than a mosquito infected at a younger age.

Finally, the interaction between temperature and aging did not shape the survival of bacterially infected mosquitoes (Fig. 4C and D and Additional File 2: Table S3). That is, the individual risk posed by warmer temperature did not alter or depend on the individual risk posed by aging, or vice versa.

### Warmer temperature accelerates the aging-dependent increase in *E. coli* infection intensity

Greater infection-related death often correlates with a heavier pathogenic load [7], so we investigated how the intensity of a bacterial infection is impacted by warmer temperature, aging, and their interaction. We reared mosquitoes at 27℃, 30℃, and 32℃, infected them with *E. coli* at 1, 5, 10, or 15 days post-eclosion, returned them to their rearing temperature, and measured the intensity of the infection 24 h later (Fig. 1). Because mosquitoes infected later in life are more likely to die, we predicted that those mosquitoes would have a higher infection intensity. Moreover, because immunosenescence proceeds more rapidly at warmer temperature for at least the melanization immune response [31], we predicted that an aging-dependent increase in infection intensity would occur earlier in life in mosquitoes maintained at a warmer temperature.

**Fig. 5:**
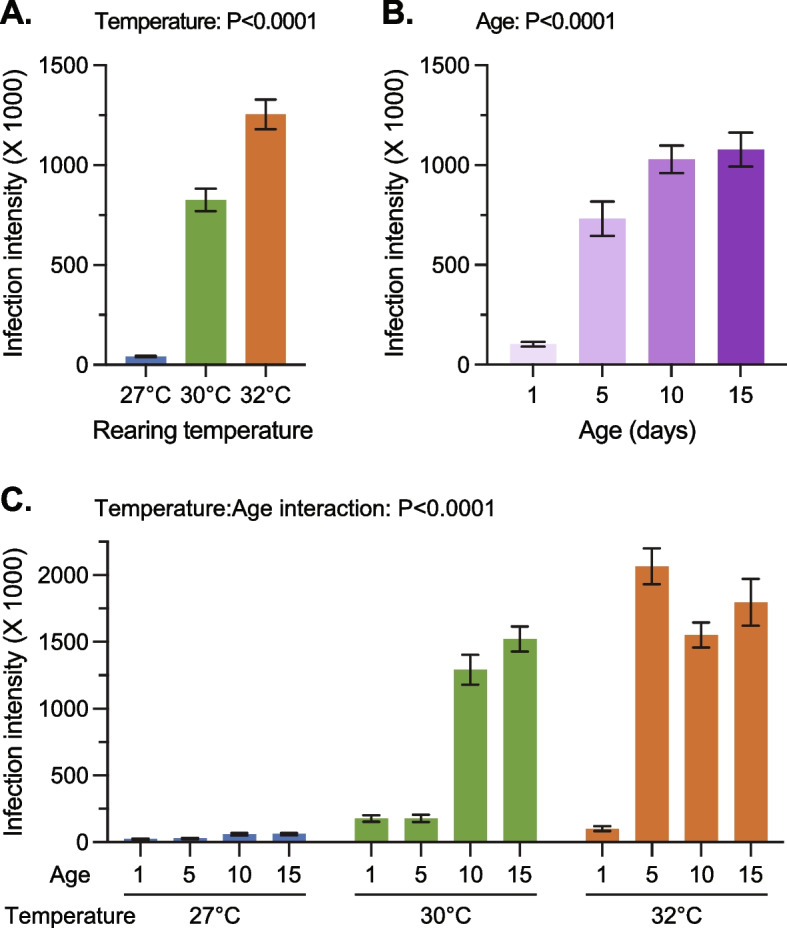
*E. coli* infection intensity increases with warmer temperature and aging, and the aging-dependent increase in intensity occurs earlier in warmer temperatures. **A** Infection intensity in mosquitoes infected at different temperatures, regardless of age. **B** Infection intensity in mosquitoes infected at different ages, regardless of the temperature. **C** Infection intensity in differently aged mosquitoes maintained at different temperatures. Column heights mark the mean and whiskers mark the standard error of the mean (SEM). Panel C shows unaggregated data, which is aggregated by temperature and age in panels A and B. Data were analyzed by a zero-inflated, negative binomial model

*E. coli* infection intensity is greatest when mosquitoes are maintained at a warmer temperature, regardless of the age when the infection was initiated (Fig. 5A). Specifically, the infection intensity at 24 h post-infection was 2000% higher in mosquitoes held at 30 °C than in mosquitoes held at 27 °C, and infection intensity was 50% higher at 32 °C than at 30 °C. The infection intensity more than doubles with each 1℃ increase in temperature (Additional File 2: Table S4), and therefore, immune efficiency declines when the temperature is warmer.

*E. coli* infection intensity is also greatest when mosquitoes are infected at an older age, regardless of temperature (Fig. 5B and Additional File 2: Table S4). Infection intensity at 24 h post-infection increased by 613% between mosquitoes infected at 1-day-old versus 5-days-old, by 41% between 5- and 10-days-old, and by 4.8% between 10- and 15-days-old. Every day of aging increases the infection intensity by 17% (Additional File 2: Table S4), and therefore, immune efficiency declines with aging.

Finally, we tested whether the effects of aging are modified by temperature, and vice versa. Temperature and aging interact to influence the *E. coli* infection intensity, as evidenced by younger mosquitoes at a warmer temperature having similar infection intensities to older mosquitoes at a cooler temperature (Fig. 5C and Additional File 2: Table S4). For example, a spike in infection intensity occurs at 5 days in mosquitoes reared at 32 °C, but it occurs instead at 10 days in mosquitoes at 30℃. Moreover, mosquitoes at 27℃ did not experience this spike in infection intensity at any age. Therefore, the aging-dependent increase in infection intensity occurs earlier and to a greater extent when the temperature is warmer.

### Temperature shapes bacterial infection intensity by affecting both mosquito and pathogen physiology

Pathogens within a mosquito may also be affected by temperature, and the *E. coli* used in these experiments grows optimally at 37 °C. Therefore, it is possible that the high infection intensity that we observed at the warmest temperature is due to an accelerated bacterial growth rate, and not factors inherent to the mosquito (i.e., the strength of the immune response). To address whether the bacterial growth rate drives the temperature phenotype, and therefore to parse the effects of warmer temperature on mosquito and bacterial physiology, we reared mosquitoes at 27 °C, 30 °C, and 32 °C, infected them with *E. coli* at 5 days of adulthood, placed all the mosquitoes at 30℃ for 24 h, and then measured infection intensity (Fig. 1). This experimental design used mosquitoes reared and maintained at different temperatures but standardized the temperature of infection. Therefore, temperature-based bacterial growth dynamics are the same across all the mosquito rearing temperatures and the experiment still captures the effect that rearing temperature has on the mosquito.

**Fig. 6:**
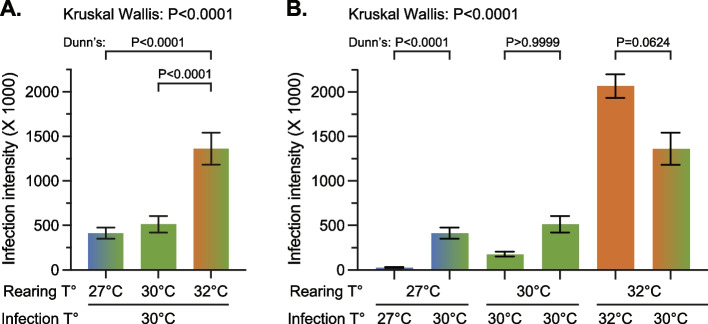
*E. coli* infection intensity is dependent on the effects of temperature on both mosquito and pathogen physiology. **A** Infection intensity in 5-day-old mosquitoes reared at 27 °C, 30 °C or 32 °C but infected at 30℃. **B** Infection intensity in 5-day-old mosquitoes reared at 27 °C, 30 °C or 32 °C, and infected at either their rearing temperature or at 30 °C. Data in panel B combines data from Fig. 5C and 6A. Column heights mark the mean and whiskers mark the standard error of the mean (SEM). Data were analyzed by Kruskal–Wallis Test followed by Dunn’s post-hoc test

Rearing temperature impacts mosquito physiology (Fig. 6A). The infection intensity in mosquitoes reared at 27℃ and 30℃ but then housed at 30 °C was similar (< 25% increase in infection intensity between 27℃ and 30℃), but the infection intensity in mosquitoes reared at 32 °C but then housed at 30 °C was 230% and 165% higher than in mosquitoes reared at 27℃ and 30℃, respectively. This demonstrates that mosquitoes reared at a warmer temperature are less capable of combating an infection, regardless of the temperature of the infection.

Temperature also impacts bacterial physiology; *E. coli* inside a mosquito replicate faster when the temperature is warmer (Fig. 6B). When 5-day-old mosquitoes were reared at 27 °C and infected for 24 h at 30 °C, the infection intensity was 1359% higher than when 5-day-old mosquitoes were reared at 27 °C and infected for 24 h also at 27 °C. Moreover, although not statistically significant, when 5-day-old mosquitoes were reared at 32 °C and infected for 24 h at 30 °C, the infection intensity was 34% lower than when 5-day-old mosquitoes were reared at 32 °C and infected for 24 h also at 32 °C. Therefore, warmer temperature affects both mosquito and bacterial physiology, increasing in infection intensity.

## Discussion

**Fig. 7:**
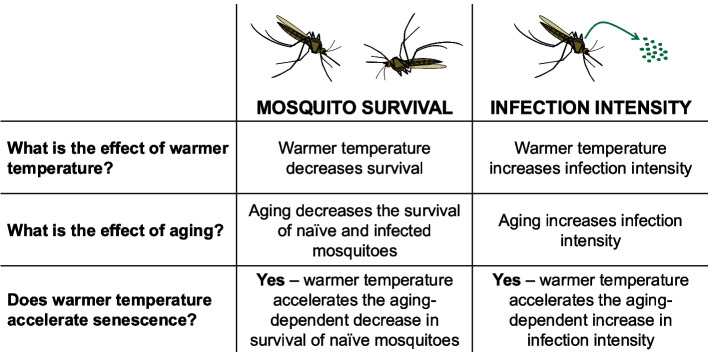
Summary of the effects of warmer temperature, aging, and their interaction on mosquito survival and *E. coli* infection intensity

A mosquito’s ability to survive depends on factors that are inherent to both the mosquito and its environment. Here, we demonstrate that survival is greatly reduced when a mosquito is infected with bacteria in the hemocoel, when the temperature is warmer, and when a mosquito ages. Temperature and aging also marginally interact to further reduce survivorship in naïve mosquitoes. Importantly, we discovered that an *E. coli* infection intensifies when the temperature is warmer and when a mosquito is older, and that warmer temperature accelerates the aging-dependent increase in infection intensity. Therefore, warmer temperature decouples chronological and physiological age, accelerating senescence (Fig. 7).

We detected an inverse relationship between warmer temperature and mosquito longevity, with mosquitoes at the warmest temperature having the shortest lifespans. This has been demonstrated in mosquitoes [14, 28], as well as in flies [45, 46], wasps [47], grasshoppers [48], and bedbugs [49]. The rate of living hypothesis predicts that ectotherms exposed to cooler temperatures have slower metabolic rates that contribute to longer lifespans [30]. Therefore, it stands to reason that the decrease in survivorship we observed at warmer temperatures is likely due to an accelerated metabolic rate and the detrimental effects associated with this. Reactions that make up the metabolic rate depend on two primary factors: (i) the concentration and fluxes of reactants and (ii) the kinetic energy of the system [17, 50]. The reactant component of the metabolic rate is directly related to the mass of the organism. Previously, using a similar experimental design, we uncovered that mosquito body size marginally decreases when the rearing temperature is increased from 27 °C to 32 °C [18], and others have shown that smaller mosquitoes have reduced longevity [51, 52]. However, because the change in body size we observed between the three temperatures is < 5% [18], we do not believe that size alone plays a meaningful role in driving longevity. The kinetic energy component of the metabolic rate is directly dependent on the temperature of the system. The body temperature of poikilotherms, like mosquitoes, varies with the temperature of the environment. The thermal optimum of an insect is often close to its critical thermal maximum, so small increases in temperature, like those experienced in this study, can cause a steep decline in fitness and performance [12, 53]. For example, warmer temperature alters nervous system function, leading to downstream endocrine system misregulation [17]. Warmer temperature also causes protein misfolding, cellular ion imbalance, mitochondrial damage, increased oxidative damage, and greater risk of desiccation, all of which decrease survival [30, 51, 53–55].

We also found that older mosquitoes are more likely to die. This makes sense, and is commonly known as an aging-dependent increase in mortality, which is characteristic of senescence [56]. This occurs in animals in general, including insects like honey bees [57], crickets [58], dragonflies [59], mosquitoes [60], and butterflies [61]. Senescence is characterized by irreversible body deterioration, a decline in the metabolic rate, lower mobility, and a weakening of immune prowess [56, 62, 63]. Therefore, older insects are usually less capable of surviving in the face of infection [7]. In this study we found that a bacterial infection at an old age disproportionately reduces survival. Specifically, mosquitoes are more likely to die when an infection is initiated at an old age than when an infection is initiated at a young age. This pattern is also observed in naïve mosquitoes but not in injured mosquitoes; injury in old age does not increase the risk of dying. Perhaps injury is governed by the fountain of youth phenotype, where stress in old age can result in greater survival [6].

Infecting young mosquitoes with *E. coli* resulted in a resiliency phenotype, where survivorship stabilized in the face of infection. Specifically, when the infection was initiated at 1-day post eclosion at 27℃, survival declined rapidly over the first 10 days, but the mosquitoes that survived past 10 days died at a similar rate as naïve mosquitoes. However, this resiliency phenotype did not occur in mosquitoes infected at an older age at 27℃. This dichotomy of the response to infection is likely explained by the damage threshold hypothesis, where insects vary their immune response strategy to suit the amount of damage caused by the pathogen [64]. This means that once infected, insects either tolerate the infection, recover from the infection, or die [64]. Our study suggests that younger mosquitoes are either more tolerant or are better at recovering from an infection compared to older ones. Therefore, younger mosquitoes are better equipped to limit the damage caused by the pathogen.

The intensity of an infection, or the pathogen burden, also impacts whether an insect tolerates an infection, recovers from an infection, or dies [64]. A higher infection intensity tends to cause greater damage and lower survival [65]. In this study we uncovered that warmer temperature increases the intensity of a hemocoelic bacterial infection and decreases longevity, which suggests that immune function declines at warmer temperatures. When isolated, each immune mechanism functions at its own temperature optimum [20, 21, 66]. For example, melanization is strongest at 18-20ºC [20, 21, 31, 66]. However, these same cool temperatures destabilize the RNAi immune response that is key to fighting viral infections [67], and expression of different immune genes varies with temperature [15, 20–22, 68, 69]. Our study demonstrates that warmer temperature decreases the additive strength of the immune mechanisms available to quell a hemocoelic bacterial infection, thus resulting in both an increase in infection intensity and a decrease in mosquito survival.

The infection intensity increases more rapidly when the infection is initiated in older mosquitoes, and this correlates with lower mosquito survivorship. Immune function declines with aging because of senescence [25]. In mosquitoes, the larval immune response is stronger than the immune response of 1-day-old adults, which is stronger than the immune response of 5-day-old adults [37]. There are several reasons for immunosenescence. As mosquitoes age, they have fewer hemocytes, and those that are present have altered phagocytic activity [7, 37, 70]. The ability to melanize pathogens also becomes less efficient with aging [26, 31]. Additionally, aging alters the rate and proportional directionality of heart contractions within the mosquito [71], which may reduce immune strength because the immune and circulatory systems are functionally integrated [72, 73].

Importantly, we discovered that temperature interacts with age such that warmer temperature accelerates the aging-dependent decline in survival and the aging-dependent increase in infection intensity. For survival, this interaction is weak and only occurs in naïve mosquitoes, where warmer temperature augments the increased risk of dying with aging and vice versa. Warmer temperature also accelerates the progression of immune senescence, causing higher infection intensity earlier in life. We previously showed that warmer temperature accelerates the aging-dependent decline in protein content of the adult mosquito [18], and accelerates the aging-dependent decline in the melanization immune response [31]. Taken altogether, we predict that other immune mechanisms and vital facets of insect physiology are similarly affected by the interaction between temperature and aging.

Changes in temperature can affect the physiology of both the insect host and the pathogen. In warmer temperatures, the extrinsic incubation period of arboviruses and *Plasmodium* becomes shorter, decreasing the length of time the pathogen needs for maturation prior to being transmitted [24, 74]. The thermal mismatch hypothesis suggests that differences in ideal temperature between hosts and pathogens leads to complex infection outcomes, where pathogens generally outperform host mechanisms under non-ideal temperature conditions, such as those seen with climate change [75]. In this study, we used live bacteria and scrutinized the contribution of temperature on both mosquito and pathogen physiology. We found that the infection intensity increased as the environmental temperature approached the optimal growth temperature for the pathogen. However, we did not see equivalent infection intensities when we reared mosquitoes at three different temperatures but conducted infections at a common temperature. Therefore, the temperature-induced changes in infection intensity are dependent on the physiology of both the mosquito and the pathogen, with the rearing temperature having a profound effect on mosquito physiology.

The experimental design of this study differs from most prior experiments testing the effect of temperature on mosquitoes. With some notable exceptions [76–78], most prior studies on temperature have reared all mosquitoes at a standard temperature (e.g., 27 °C) and after treatment transferred them to various experimental temperatures, thus introducing a temperature shift [20, 21, 24, 28, 68, 69]. This distinction is important because acclimatization impacts the metabolic rate and physiology of insects [78, 79]. Our approach captured the effects of temperature that arise from the larval, pupal, and adult experience, which is salient because the experience of both immatures and adults impacts the ability of an adult mosquito to survive and respond to infection [76, 77, 80, 81].

The present study only examined non-blood fed mosquitoes, but female anautogenous mosquitoes like *A. gambiae* take a blood meal to obtain the nutrients needed to produce eggs [82]. For humans and other animals, blood feeding by mosquitoes is consequential because it can transmit blood-borne pathogens [82]. Based on our findings, future studies should assess how blood feeding affects how temperature and aging interact to shape survival and infection intensity. Blood feeding on a human or another mammal temporarily raises the temperature of the mosquito, and this process modifies the immune system by, for example, increasing the number of hemocytes and affecting the expression of immune genes [83–85]. Blood feeding also triggers the production of the eggs that are essential for the next generation, and the interaction between temperature and aging may result in trade-offs between survival and reproduction, such as terminal investment, reproductive restraint, or reproductive senescence [86–88].

## Conclusions

This study demonstrates that warmer environmental temperature accelerates aging in mosquitoes, altering both longevity and infection outcomes. Additionally, this study disentangles interactive effects between temperature and senescence in poikilothermic ectotherms, demonstrating that insect models must account for how environmental factors shape the internal processes of pollinators, agricultural pests, and disease vectors when predicting the beneficial and detrimental effects of insect populations to human survival and wellbeing.

## Supplementary Information

Additional file 1: File S1. Data collected during this study.

Additional file 2: Table S1. Cox nonproportional hazards with weighted estimation statistical information for Figure 3. Table S2 Median survival and maximum lifespan summary table. Table S3 Cox nonproportional hazards with weighted estimation (stratified by immune treatment) statistical information for Figure 4. Table S4 Zero-inflated negative binomial regression model statistical information for Figure 5.

Additional file 3: Figure S1 The survival of naïve mosquitoes declines with warmer temperature, aging, and their interaction. Figure S2 The survival of injured mosquitoes declines with warmer temperature. Figure S3 The survival of infected mosquitoes declines with warmer temperature and aging.

## Data Availability

All data generated or analyzed during this study are included in this published article and its supplementary information files.
