# Supplementary material for: Warmer environmental temperature accelerates aging in mosquitoes, decreasing longevity and worsening infection outcomes
Source: Immun Ageing. 2024 Sep 11;21:61. doi: 10.1186/s12979-024-00465-w (PMC11389126; doi:10.1186/s12979-024-00465-w)
Supplement: Supplementary file 2 — Additional file 2: Table S1. Cox nonproportional hazards with weighted estimation statistical information for Figure 3. Table S2 Median survival and maximum lifespan summary table. Table S3 Cox nonproportional hazards with weighted estimation (stratified by immune treatment) statistical information for Figure 4. Table S4 Zero-inflated negative binomial regression model statistical information for Figure 5. [file 12979_2024_465_MOESM2_ESM.pdf]

Warmer environmental temperature accelerates aging in mosquitoes, decreasing longevity and worsening infection outcomes

Jordyn S. Barr, Lindsay E. Martin, Ann T. Tate, & Julián F. Hillyer
Department of Biological Sciences, Vanderbilt University, Nashville, TN, USA
julian.hillyer@vanderbilt.edu

***Immunity & Ageing*, 2024**

Table S1. Cox non-proportional hazards with weighted estimation statistical information for Figure 3.

Table S1					
Mosquito Survival: 4 Immune Treatments at 3 Temperatures and 4 Ages					
**Cox Non-proportional Hazards with Weighted Estimation**					
**survival(start, age of death + 1, censor) ~ temperature * age + strata(treat) + frailty(block)**					
**Infection Stratified Model**	**Coefficient**	**Hazard Ratio**	**Lower 95%**	**Upper 95%**	**P-value**
Injured	0.2337	1.2633	1.1808	1.3516	**<0.001**
E. coli infected	1.1335	3.1066	2.9189	3.3063	**<0.001**
M. luteus infected	0.6018	1.8254	1.6828	1.9802	**<0.001**
Temperature	0.3286	1.3890	1.3542	1.4248	**<0.001**
Age	0.1608	1.1745	1.0777	1.2800	**<0.001**
Temperature:Age	-0.0025	0.9975	0.9945	1.0004	0.0932

Warmer environmental temperature accelerates aging in mosquitoes, decreasing longevity and worsening infection outcomes

Jordyn S. Barr, Lindsay E. Martin, Ann T. Tate, & Julián F. Hillyer
Department of Biological Sciences, Vanderbilt University, Nashville, TN, USA
julian.hillyer@vanderbilt.edu

Table S2. Median survival and maximum lifespan summary table. (Part A)

Table S2				
Mosquito Survival: 4 Immune Treatments at 3 Temperatures and 4 Ages				
**Median Survival and Maximum Lifespan Summary**				
Naïve	1 Day		5 Days	
	Median Survival (Days)	Max Lifespan Survival (Days)	Median Survival (Days)	Max Lifespan Survival (Days)
27°C	21	38	21	38
30°C	12	29	12	26
32°C	8	24	10	19
Injured	1 Day		5 Days	
	Median Survival (Days)	Max Lifespan Survival (Days)	Median Survival (Days)	Max Lifespan Survival (Days)
27°C	20	38	18	40
30°C	11	30	10	20
32°C	7	20	9	17
E. coli-infected	1 Day		5 Days	
	Median Survival (Days)	Max Lifespan Survival (Days)	Median Survival (Days)	Max Lifespan Survival (Days)
27°C	9	44	11	34
30°C	4	17	7	15
32°C	3	16	7	17
M. luteus-infected	1 Day		5 Days	
	Median Survival (Days)	Max Lifespan Survival (Days)	Median Survival (Days)	Max Lifespan Survival (Days)
27°C	20	37	17	34
30°C	14	30	11	33
32°C	4	15	7	17

Table S2. Median survival and maximum lifespan summary table. (Part B)

Table S2				
Mosquito Survival: 4 Immune Treatments at 3 Temperatures and 4 Ages				
**Median Survival and Maximum Lifespan Summary**				
Naïve	10 Days		15 Days	
	Median Survival (Days)	Max Lifespan Survival (Days)	Median Survival (Days)	Max Lifespan Survival (Days)
27°C	17	33	22	37
30°C	14	26	17	24
32°C	13	17	N/A	N/A
Injured	10 Days		15 Days	
	Median Survival (Days)	Max Lifespan Survival (Days)	Median Survival (Days)	Max Lifespan Survival (Days)
27°C	19	36	19	37
30°C	12	24	17	26
32°C	11	22	N/A	N/A
E. coli-infected	10 Days		15 Days	
	Median Survival (Days)	Max Lifespan Survival (Days)	Median Survival (Days)	Max Lifespan Survival (Days)
27°C	12	22	18	32
30°C	11	16	16	19
32°C	11	13	N/A	N/A
M. luteus-infected	10 Days		15 Days	
	Median Survival (Days)	Max Lifespan Survival (Days)	Median Survival (Days)	Max Lifespan Survival (Days)
27°C	17.5	35	17	38
30°C	14	25	16	20
32°C	11	14	N/A	N/A

Warmer environmental temperature accelerates aging in mosquitoes, decreasing longevity and worsening infection outcomes

Jordyn S. Barr, Lindsay E. Martin, Ann T. Tate, & Julián F. Hillyer
Department of Biological Sciences, Vanderbilt University, Nashville, TN, USA
julian.hillyer@vanderbilt.edu

Table S3. Cox non-proportional hazards with weighted estimation (stratified by immune treatment) statistical information for Figure 4.

Table S3					
Mosquito Survival: 4 Immune Treatments at 3 Temperatures and 4 Ages					
**Cox Non-proportional Hazards with Weighted Estimation (Stratified by Treatment)**					
**survival(start, age of death + 1, censor) ~ temperature * age + frailty(block)**					
Naïve	**Coefficient**	**Hazard ratio**	**Lower 95%**	**Upper 95%**	**P-value**
Temperature	0.4238	1.5278	1.4679	1.5900	**<0.001**
Age	0.2692	1.3090	1.1456	1.4955	**<0.001**
Temperature:Age	-0.0078	0.9923	0.9877	0.9968	**<0.001**
Injured	**Coefficient**	**Hazard ratio**	**Lower 95%**	**Upper 95%**	**P-value**
Temperature	0.2363	1.2666	1.2024	1.3343	**<0.001**
Age	-0.0185	0.9817	0.8206	1.1744	0.8396
Temperature:Age	0.0042	1.0042	0.9980	1.0103	0.1833
E.coli-infected	**Coefficient**	**Hazard ratio**	**Lower 95%**	**Upper 95%**	**P-value**
Temperature	0.2856	1.3306	1.2547	1.4111	**<0.001**
Age	0.3337	1.3961	1.0800	1.8046	**0.0108**
Temperature:Age	-0.0051	0.9949	0.9861	1.0037	0.2552
M. luteus-infected	**Coefficient**	**Hazard ratio**	**Lower 95%**	**Upper 95%**	**P-value**
Temperature	0.3778	1.4591	1.3692	1.5550	**<0.001**
Age	0.2777	1.3201	1.0780	1.6167	**0.0072**
Temperature:Age	-0.0049	0.9951	0.9883	1.0020	0.1619

Warmer environmental temperature accelerates aging in mosquitoes, decreasing longevity and worsening infection outcomes

Jordyn S. Barr, Lindsay E. Martin, Ann T. Tate, & Julián F. Hillyer
Department of Biological Sciences, Vanderbilt University, Nashville, TN, USA
julian.hillyer@vanderbilt.edu

Table S4. Zero-inflated negative binomial regression model statistical information for Figure 5.

Table S4							
Infection Intensity: *E. coli* Infection at 3 Temperatures and 4 Ages; Infection at 3 Temperatures							
**Zero-inflated Negative Binomial Regression Model**							
**CFUs ~ temperature + age + temperature*age | 1**							
	**Estimate (log scale)**	**Std. Error**	**Bootstrapped Estimate (response scale)**	**Lower 95% (bcaLL)**	**Upper 95% (bcaUL)**	**P-value**	**Data Interpretation**
Temperature	0.7089	0.0243	2.032	1.9297	2.1216	**<0.0001**	With every degree increase in temperature, the infection intensity more than doubles, given that age is held constant at the average.
Age	0.1585	0.0095	1.1717	1.1508	1.1940	**<0.0001**	With every day increase in age, the infection intensity increases by 17%, given that temperature is held constant at the average .
Temperature:Age	0.0240	0.0057	1.0243	1.0116	1.0388	**<0.0001**	Temperature and age interact significantly to increase infection intensity.
