# Supplementary material for: Warmer environmental temperature accelerates aging in mosquitoes, decreasing longevity and worsening infection outcomes
Source: Immun Ageing. 2024 Sep 11;21:61. doi: 10.1186/s12979-024-00465-w (PMC11389126; doi:10.1186/s12979-024-00465-w)
Supplement: Supplementary file 3 — Additional file 3: Figure S1 The survival of naïve mosquitoes declines with warmer temperature, aging, and their interaction. Figure S2 The survival of injured mosquitoes declines with warmer temperature. Figure S3 The survival of infected mosquitoes declines with warmer temperature and aging. [file 12979_2024_465_MOESM3_ESM.pdf]

Warmer environmental temperature accelerates aging in mosquitoes, decreasing longevity and worsening infection outcomes

Jordyn S. Barr, Lindsay E. Martin, Ann T. Tate, & Julián F. Hillyer
Department of Biological Sciences, Vanderbilt University, Nashville, TN, USA
julian.hillyer@vanderbilt.edu

***Immunity & Ageing*, 2024**

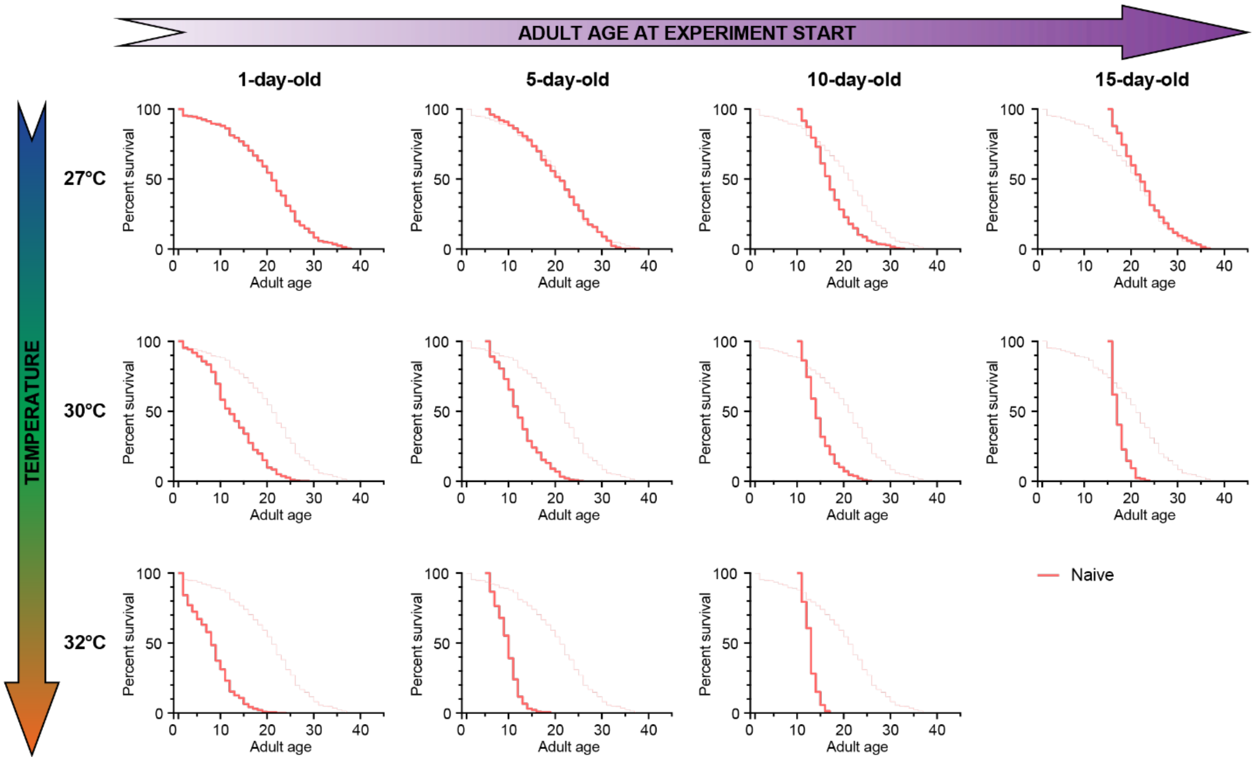

Figure S1. The survival of naïve mosquitoes declines with warmer temperature, aging, and their interaction. Kaplan-Meier curves show the survival of naïve mosquitoes for each temperature and age combination. The curves are organized from left to right by increasing age and from top to bottom by increasing temperature. The survival of 1-day-old naïve mosquitoes at 27°C is presented as a faint line in all panels for comparison purposes.

Warmer environmental temperature accelerates aging in mosquitoes, decreasing longevity and worsening infection outcomes

Jordyn S. Barr, Lindsay E. Martin, Ann T. Tate, & Julián F. Hillyer
Department of Biological Sciences, Vanderbilt University, Nashville, TN, USA
julian.hillyer@vanderbilt.edu

***Immunity & Ageing*, 2024**

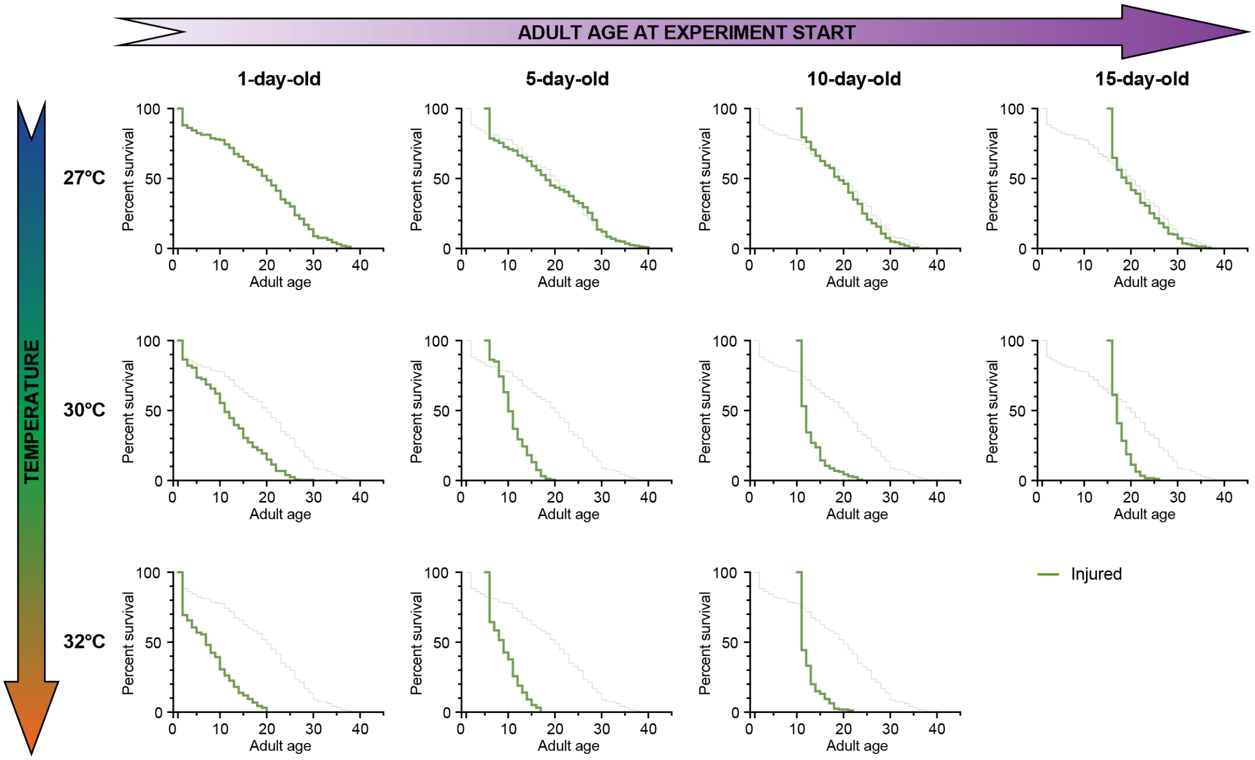

Figure S2. The survival of injured mosquitoes declines with warmer temperature. Kaplan-Meier curves show the survival of injured mosquitoes for each temperature and age combination. The curves are organized from left to right by increasing age and from top to bottom by increasing temperature. The survival of 1-day-old injured mosquitoes at 27°C is presented as a faint line in all panels for comparison purposes.

Warmer environmental temperature accelerates aging in mosquitoes, decreasing longevity and worsening infection outcomes

Jordyn S. Barr, Lindsay E. Martin, Ann T. Tate, & Julián F. Hillyer
Department of Biological Sciences, Vanderbilt University, Nashville, TN, USA
julian.hillyer@vanderbilt.edu

***Immunity & Ageing*, 2024**

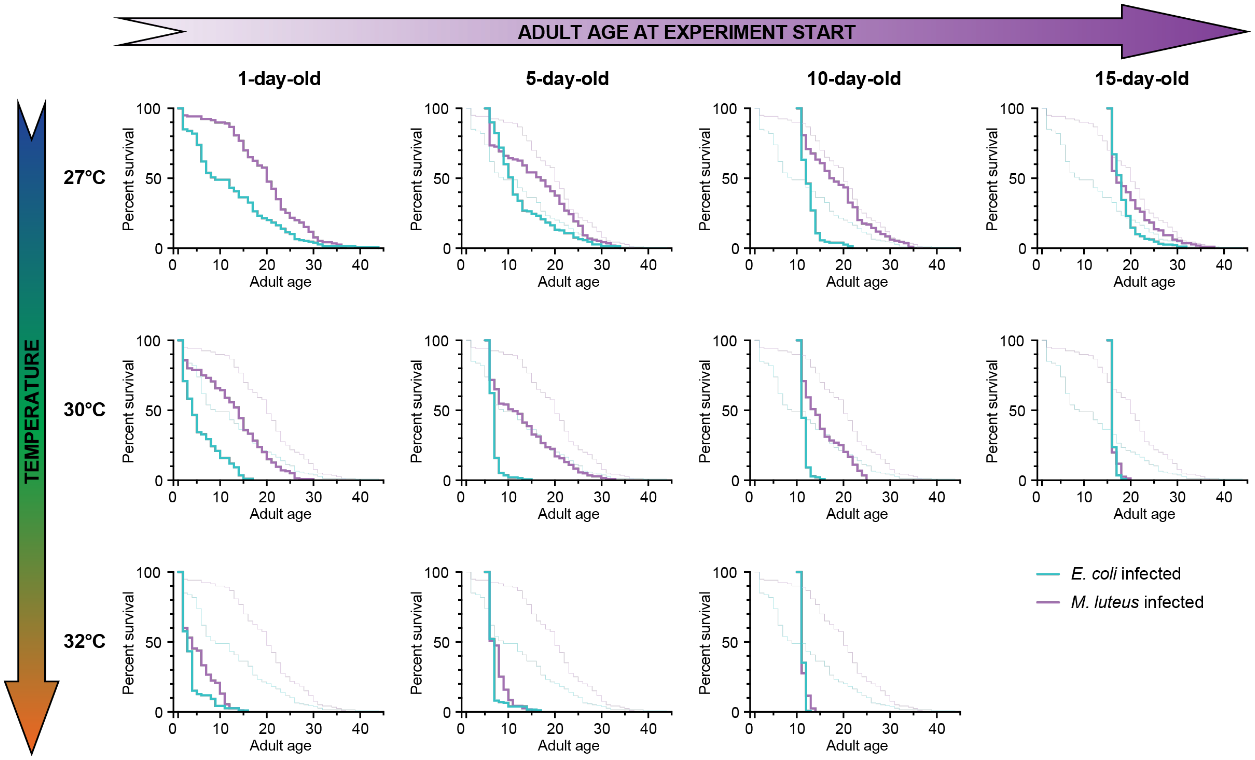

Figure S3. The survival of infected mosquitoes declines with warmer temperature and aging. Kaplan-Meier curves show the survival of *E. coli*- and *M. luteus*-infected mosquitoes for each temperature and age combination. The curves are organized from left to right by increasing age and from top to bottom by increasing temperature. The survival of 1-day-old *E. coli*- and *M. luteus*-infected mosquitoes at 27°C is presented as two faint lines in all panels for comparison purposes.
